# Pro-Oncogenic Transcription Factors BACH1 and Nrf2 Associate with Cytoplasmic Biomolecular Condensates of GFP-MxA (Myxovirus Resistance Protein A) in Oral Cancer Cells

**DOI:** 10.3390/cells15110982

**Published:** 2026-05-26

**Authors:** Pravin B. Sehgal, Huijuan Yuan

**Affiliations:** 1 Department of Cell and Molecular Physiology, New York Medical College, Valhalla, NY 10595, USA; 2 Department of Medicine, New York Medical College, Valhalla, NY 10595, USA

**Keywords:** pathogenesis of oral cancer, environmental stresses and carcinogens, anatomical sites of oral cancer, biomolecular condensates, human myxovirus resistance protein (MxA/Mx1), BACH1, Nrf2, heme oxygenase 1 (HO1), antioxidant condensates

## Abstract

**Highlights:**

**What are the main findings?**

- An unbiased proteomics study of biomolecular condensates of cytoplasmic human GFP-MxA in oral cancer cells revealed the inclusion of transcription factors BACH1 and Nrf2. Additionally, these condensates also included heme oxygenase 1 (HO1).
- GFP-MxA and Nrf2 in condensates were rapidly disassembled and then reassembled into new structures by exposing oral cancer cells to hypotonicity and then isotonicity. Moreover, these condensates were disassembled by sulforaphane.

**What are the implications of the main findings?**

- The data define a class of antioxidant condensates responsive to environmental stresses.
- Such stress-responsive biomolecular condensates which include transcription factors may contribute to the pathogenesis of oral carcinoma.

**Abstract:**

Biomolecular condensates in the cytoplasm and nucleus contribute to carcinogenesis through aberrant signaling by assorted transcription factors and fusion oncoproteins. Oral cancer, which is highly prevalent worldwide, frequently occurs in a U-shaped “high-risk” zone (floor of mouth, side of tongue, and anterior fauces) which forms the path of liquid transit through the mouth. We previously reported that environmental stresses of saliva-like hypotonicity and beverage-like temperature changes triggered cycles of disassembly/reassembly of biomolecular condensates of GFP-tagged human myxovirus resistance protein (MxA; alias Mx1) in oral cancer cells. In the present study, we identified some of the constituents of GFP-MxA cytoplasmic condensates in oral cells. These condensates were isolated from interferon (IFN)-λ1-treated GFP-MxA expressing OECM1 human oral cancer cells using magnetic bead-based immunoisolation. Unbiased peptide identification confirmed the presence of MxA/Mx1 peptides; however, the strongest intensity was for the BACH1 transcription factor family. Immunofluorescence analyses confirmed the association of BACH1 and the family member Nrf2 with cytoplasmic human GFP-MxA condensates. Moreover, GFP-BACH1 and GFP-Nrf2 colocalized with cytoplasmic human HA-MxA condensates in transiently transfected OECM1 cells. Western blot assays confirmed the presence of BACH1 and Nrf2 proteins in complexes isolated using anti-MxA pAb. As much as BACH1 and Nrf2 regulate oxidative stress response genes, it was remarkable that immunofluorescence assays revealed the presence of heme oxygenase 1 (HO1)—a downstream redox regulator—in GFP-MxA condensates. However, these condensates were devoid of p62, KEAP1 and Cul3. In terms of aberrant function, in live cells, the Nrf2 transcription factor underwent rapid disassembly and reassembly cycles driven by saliva-like hypotonicity, and was also disassembled by sulforaphane. The data highlight the unexpected intersections in oral cells between MxA condensates and BACH1, Nrf2 and HO1—proteins well known to be involved in pathways regulating cellular responses to environmental and oxidative stresses, antiviral defense, oral epithelial dysplasia, and cancer progression and metastases.

## 1. Introduction

Oral cancer, which is prevalent worldwide, has a significant 5-year mortality of 45–50% [1,2,3,4]. Insights into the pathogenesis of this cancer have been typically obtained from patients already diagnosed with cancer, followed by the physician’s attempt to unravel the patient’s history [1,2,3,4,5,6,7,8]. Such studies have uncovered preventable causes such as tobacco or betel nut/pan leaf chewing, smoking and alcohol consumption using products usually of local manufacture containing “addictive carcinogens” (e.g., arecoline in betel nut) [1,2,3,4,5,6,7,8,9]. In these instances, the “exposome” includes the buccal mucosa of the cheek or the lower lip (where the quid is held) and the floor of the mouth along the path of liquid transit [1,3]. Overt causes also include chronic mechanical irritation of the mucosa due to dental prostheses (e.g., ill-fitting dentures) [1]. Long-term consumption of beverages at or above 150 °F has also been associated with an increased cancer risk, as for the upper esophagus [10,11].

**Figure 3:**
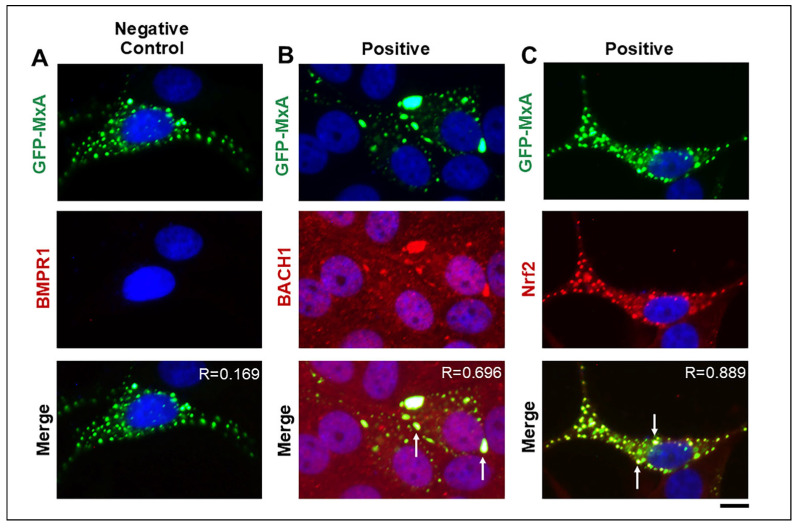
Immunofluorescence analyses for the association of some candidate proteins from Panel 1D with GFP-MxA condensates. OECM1 cultures expressing GFP-MxA were fixed two days after transfection and evaluated for the presence of BACH1 or Nrf2 in the GFP-MxA condensates in immunofluorescence assays. Panel (**A**) represents a negative control using an irrelevant anti-BMPR1 pAb. Panels (**B**,**C**) represent data for BACH1 and Nrf2 respectively. White arrows point to GFP condensates containing BACH1 or Nrf2 by immunofluorescence.

Importantly, a significant subset of patients with oral cancer reveals no overt causes [4]. In a pioneering study of such cases published 50 years ago, Mashberg and Myers identified a U-shaped “high-risk” zone comprising the floor of the mouth, especially around the papilla of the submandibular duct, the side of the tongue, the anterior pillar of the fauces and the retromolar region [4]. They reported that 97% of 207 intraoral malignant lesions detected “prospectively” in such asymptomatic patients occurred in this “high-risk” zone (especially see Figure 3a,b in reference [4]). The authors called for “increased scrutiny” of the underlying cell biology of the epithelium of this region [4]. This region comprises the pathway of liquid (and beverage) flow through the mouth [3,4,12,13]. The cellular basis for the anatomic localization of oral cancer occurrence in this high-risk zone of cancer development in the mouth in the absence of any overt causes remains incompletely understood [1,2,3,4]. Investigators have considered changes in the oral microbiome, including bacterial, fungal and viral agents (including papilloma and the Epstein–Barr virus [2,3,7,8,9], and citations therein), enhanced heme oxygenase 1 (HO1). Molecular drivers such as the E6 protein of human papilloma virus, pro-oncogenic p-STAT3 transcription factor, and mutations in p53 and Rb have been considered [2,3,14,15,16]) have received attention. The present study addresses novel discoveries in biomolecular condensate biology in cells likely applicable to this high-risk zone [17,18,19,20,21,22,23,24,25].

Liquid–liquid phase-separated (LLPS) biomolecular condensates in the cytoplasm and nucleus contribute to carcinogenesis through dysregulation of signaling pathways, and aberrant function of fusion oncoproteins and transcription factors [17,18,19,20,21,22,23,24,25]. Indeed, condensate disassembly and spontaneous reassembly during cellular stress responses, the regulation of transcription and translation by condensate droplets, and in cancer pathogenesis, in innate and adaptive immunity, cytokine signaling, viral replication and antiviral mechanisms, and the targeting of condensates by cancer therapeutic agents have been highlighted by numerous investigators [17,18,19,20,21,22,23,24,25].

Our previous studies have focused on the dynamic alterations of the structure and biology of cytoplasmic condensates of interferon (IFN)-induced human antiviral “myxovirus resistance protein” (MxA alias Mx1), a dynamin-family large GTPase (approx. 60 kDa [19,24,25,26,27,28]. Cytoplasmic human MxA and nuclear murine Mx1 exhibit antiviral activity towards influenza A virus (IAV) and additional RNA and DNA viruses [27]. We previously discovered that membraneless cytoplasmic human MxA condensates showed rapid disassembly in 1–2 min in cells exposed to hypotonic medium, and rapid reassembly (also in 1–2 min) in cells shifted back to isotonic medium [24,25,27,28]. Saliva exiting the submandibular duct (in the middle of the high-risk zone of oral cancer development, Ref. [4]) is hypotonic (approximately 100 mOsm, i.e., approximately one-third tonicity compared to plasma) (Refs. [3,4,12,13], and citations therein). Thus, we investigated whether hypotonicity- and beverage-like temperature-driven changes in condensate cell biology in the mouth might drive pathogenesis of oral cancer. Since MxA is known to be constitutively present in normal human gingiva [29], we used GFP-MxA as a reporter of condensate dynamics in oral cells [25,28,30]. Based on our previous data about the rapid and reversible dynamics of MxA condensates in oral epithelial cells [25,28,30], we considered a novel hypothesis for the anatomic localization of oral cancer in the U-shaped “high-risk” zone in the mouth: that dysfunction of diverse biomolecular condensates in oral cells along the beverage transit pathway through the mouth due to repetitive tonicity and temperature stresses that might underlie a prooncogenic progression leading to oral cancer [25].

In the present study, we identified some of the constituents of GFP-MxA cytoplasmic condensates in oral cancer cells by isolating GFP-MxA structures from prefixed interferon (IFN)-λ1-treated GFP-MxA expressing OECM1 human oral cancer cells using magnetic-bead-based immunoisolation, followed by unbiased peptide/protein family identification. The new data highlight the unexpected discovery that MxA condensates contain the transcription factors BACH1 (BTB and CNC homology 1) and Nrf2 (nuclear factor erythroid 2-related factor 2), and also their downstream responder protein heme oxygenase 1 (HO1). Taken together, these proteins are well known to be involved in cellular responses to environmental and oxidative stresses, antiviral defense, oral epithelial dysplasia, and cancer progression and metastases [2,31,32,33,34,35,36,37,38,39,40,41,42]. The new data provide support for the hypothesis that the tonicity- and temperature-driven dynamic repetitive changes (disassembly/reassembly) of condensates involving prooncogenic transcription factors in oral cells located along the liquid transit pathway through the mouth might contribute to carcinogenesis.

## 2. Materials and Methods

### 2.1. Cells and Cell Culture

Human oral carcinoma cell line OECM1 was purchased from Millipore-Sigma (St. Louis, MO, USA), while the human lung adenocarcinoma cell line A549 was obtained from the American Type Culture Collection (Manassas, VA, USA). The respective cell lines were grown in DMEM (Corning Cat. No. 10-013-CV, with glutamine, Na-pyruvate and high glucose) supplemented with 10% *v*/*v* fetal bovine serum (FBS; Gibco, Grand Island, NY, USA) in T25 flasks, 35 mm dishes without or with cover-slip bottoms [24,28,30].

### 2.2. Plasmids and Transient Transfection

The GFP (1-248)-tagged full-length human MxA and GFP-murine Mx1 constructs were gifts from Dr. Jovan Pavlovic (University of Zurich, Zurich, Switzerland) [24,43]. HA = tagged MxA was a gift from Dr. Otto Haller (University of Freiburg, Freiburg, Germany) [24]. The EGFP-tagged human BACH1 construct was purchased from Genescript (Piscataway, NJ, USA) while the EGFP-tagged human Nrf2 construct was obtained from Addgene, Watertown, MA, USA. Transient transfections were carried out using the Polyfect reagent (Qiagen, Germantown, MD, USA) and the manufacturer’s protocol [24,28,30].

### 2.3. Fluorescence Imaging

Live-cell imaging of GFP-MxA structures in transiently transfected cells was carried out in cells grown in 35 mm plates using the upright Zeiss AxioImager 2 (Carl Zeiss, Inc., Hawthorne, NY, USA) as described earlier [24,28,30]. Moreover, confocal imaging, including z-stack imaging, was carried out by growing cells in 35 mm plates with glass cover-slip-bottoms, formaldehyde fixation followed by imaging with a 63× oil immersion objective and Zeiss LSM980 confocal microscope (Carl-Zeiss Inc., Hawthorne, NY, USA).

### 2.4. Phase Transition Experiments

GFP-MxA-expressing cells two days after transient transfection were exposed to fresh full growth medium or phosphate-buffered saline (PBS) at 37 °C and imaged (the “0 min” time). Subsequently the cultures were shifted to 1:3 or 1:4 dilution of the medium (adjusted with water) or hypotonic ELB (50 mOsm) for 4–5 min and then either fixed using 4% paraformaldehyde (PFA) (for 1 h at room temperature in the hypotonic buffer, or live cultures switched to full strength medium or to PBS for 5 min, then imaged, and then fixed using 4% PFA in full strength medium. Fluorescence was imaged as summarized above [24,28,30].

### 2.5. Magnetic-Bead-Based Immunoisolation of GFP-MxA Condensates and Unbiased Proteomics

**Figure 1:**
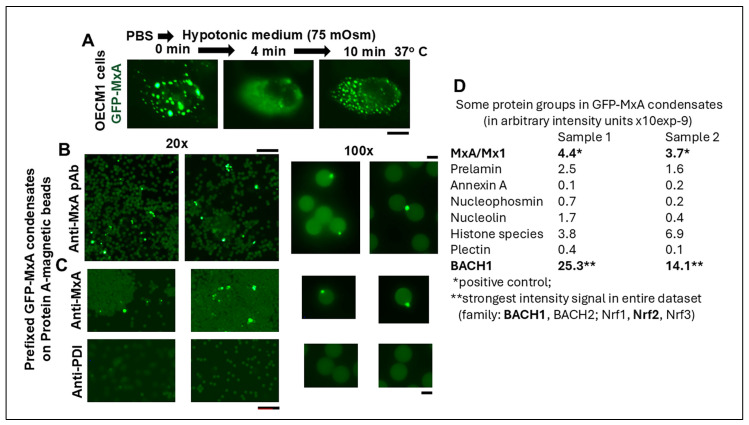
Panel (**A**), rapid disassembly of GFP-MxA condensates in oral cancer cells exposed to saliva-like hypotonic medium (1:4 dilution of full medium) and *spontaneous* reassembly into new condensates even in the continued presence of hypotonic medium. Scale bar = 10 µm. Panels (**B**,**C**), immunoisolation of GFP-MxA condensates from prefixed (2% PFA, 10 min) A549 cells subjected to pullout using Protein A-Dynabeads and the indicated antibodies. Scale bars = 10 µm in the 20× panels, and = 5 µm in the 100× panels. Panel (**D**), duplicate samples of GFP-MxA condensates immunoisolated from sonicates of prefixed transiently transfected and IFN-λ1-treated (50 ng/mL, 2 days) OECM1 cells were subjected to unbiased proteomics evaluation (following tryptic digestion, mass spectroscopy and unbiased peptide identification by comparison with human peptide databases). Intensity (in arbitrary units) represents the summed-up eXtracted Ion Current (XIC) of all isotopic clusters associated with the identified amino acid sequence. Panel (**D**) represents the top 8 entries from >200 protein families identified.

We showed earlier that, as with stress granules [44], the integrity of GFP-MxA condensates requires that the cells remain intact [24,28,30]. Thus, we used the methods developed by Parker and colleagues to isolate stress granules from PFA prefixed cells using magnetic-bead-based immunoisolation followed by trypsin digestion, mass-spectroscopy and unbiased peptide identification [44]. Briefly GFP-MxA transfected cells in 35 or 90 mm culture dishes were additionally treated with IFN-λ1 (50 ng/mL) for 2 days, fixed at room temperature using 2% PFA in PBS for 10 min, washed with PBS, while the PFA was quenched with 1 M glycine in ELB for 10 min, the cultures washed in cold PBS and then harvested by scraping into cold “GG” buffer (1 mL/plate) (20 mM TrisHCl, pH 7.4, 100 mM NaCl, 1 mM EDTA, 0.1% SDS, 0.5% Triton X-100, 1 mM PMSF). The suspension was sonicated for 45–60 s, the debris centrifuged (1000 rpm, 5 min) and droplets of the supernatant were verified for the presence of GFP-MxA condensates by fluorescence microscopy. Aliquots of the supernatant fraction were then mixed with anti-MxA pAb or irrelevant pAb (such as anti-protein disulfide isomerase, PDI, pAb), mixed with 50 µL of a GG-buffer, washed, diluted 1:5 with Protein A-Dynabead suspension (30 mg/mL original suspension, Cat. 2848440, Invitrogen, Baltics, Norway), followed by incubation overnight at 4 °C. The beads were then washed 4× using cold PBS and magnetic separation; the pellets were air-dried and used for tryptic digestion, mass-spectroscopy and unbiased peptide identification by comparison with a human peptide database by Creative Proteomics (Shirley, NY, USA). The signal intensity of respective peptide families was expressed in arbitrary intensity units (Figure 1D).

### 2.6. Immunofluorescence Assays

The most intense 8–10 such peptide family identifications were tested for association with GFP-MxA condensates in PFA-fixed (4%, 1 h) transfected cells using two-color immunofluorescence assays as described earlier [24,28,30]. Fluorescence data were collected as mentioned briefly in Section 2.3 above. Between 5 and 20 cells were imaged per variable. Numerical analyses of colocalization were carried out using Image J FIJI version x86.64 and expressed in terms of Pearson’s correlation coefficient R, indicated on respective images. All scale bars in the figures equal 10 µm, unless indicated otherwise.

### 2.7. Western Blotting

Aliquots of prefixed or unfixed cell extracts prepared as outlined above in Section 2.5 were used to immunoisolate complexes reactive with respective antibodies (overnight incubation with Protein A-Dynabeads), followed by washing 3–4× with PBS and resuspension in 2× Laemmli sample loading buffer. Electrophoreses were carried out using 4–20% Mini-PROTEAN TGX precast gels (BioRad, Hercules, CA, USA) or 4–15% SMOBio QPAGE (Stellar Scientific, Baltimore, MD, USA) precast gels, followed by Western blotting [24,28,30]. Proteins were displayed using relevant primary rabbit antibodies and CF488A-tagged donkey anti-rabbit IgG from Biotium Inc. (Cat. 20015) (Freemont, CA, USA) with the assistance of an iBright1500 Invitrogen Imaging system (Thermo-Fisher Scientific, Tewkesbury, MA, USA).

### 2.8. Chemicals and Antibody Reagents

D,L-Sulforaphane (SFN) (Cat. No. 574215—25 mg) was purchased from Millipore-Sigma Inc. (Burlington, MA, USA) and ML385 (Cat. No. 6243) was purchased from Tocris Biosciences (Bristol, UK). Rabbit pAb to human MxA (H-285) (ab-95926) was purchased from Abcam Inc. (Cambridge, MA, USA). Rabbit pAbs to human BACH1 (Cat. No. 14018-1-AP), to human HO1 (Cat. No. 10701-1-AP), human Nrf2 (Cat. No. 16396-1-AP), to human p62 (Cat. No. 18420-1-AP), to human KEAP1 (Cat. No. 10503-2-AP) and to human Cul3 (Cat. No. 1107-1-AP) were purchased from Proteintech Group Inc., (Rosemont, IL, USA). Rabbit pAbs to human protein disulfide isomerase (PDI; Cat. No. sc-20132) and to clathrin heavy chain (CHC; Cat. No. sc-9069) were obtained from Santa Cruz Biotechnology Inc., (Dallas, TX, USA). Respective AlexaFluor 488- and AlexaFluor 594-tagged secondary donkey antibodies to rabbit (A-11008 and A-11012) or mouse (A-21202 and A-21203) IgG were from Invitrogen Molecular Probes (Eugene, OR, USA) for immunofluorescence assays, or CF488-tagged donkey anti-rabbit IgG from Biotium Inc. (Cat. 20015) (Fremont, CA, USA) for Western blots.

## 3. Results

### 3.1. Isolation of GFP-MxA Condensates and Unbiased Peptide Identification

As reported by us earlier [25,30], live-cell fluorescence imaging of transiently expressed wild-type GFP-MxA in OECM1 cells exposed to hypotonic buffer (75–100 mOsm, approximately corresponding to saliva-like/beverage-like hypotonicity) confirmed the rapid disassembly of GFP-MxA condensates within 2–4 min, followed by spontaneous reassembly of condensates by 7–10 min even when cells were continuously kept under hypotonic conditions (Figure 1A). Moreover, gently permeabilizing cells with 0.05% saponin led to rapid disassembly of GFP-MxA condensates [24]. Thus, as with the methods of Parker and colleagues for isolation of stress granules [44], it was necessary to prefix cells with 2% PFA for 10 min prior to cell harvesting and sonication. Preliminary observations in transfected A549 cells confirmed that such prefixation allowed GFP-MxA structures to remain intact. The sonicated supernate was clarified by low-speed centrifugation and GFP-MxA condensates were immunoisolated using anti-MxA pAb and Protein A-Dynabeads using magnetic pullout. Figure 1B shows low and high-magnification images of washed pellets of such isolates, confirming the association of GFP-MxA condensates with Dynabeads. Figure 1C confirms that such isolation was anti-MxA immune specific and did not occur with the irrelevant control pAb against protein disulfide isomerase (PDI). Subsequently, duplicate samples of anti-GFP-MxA condensate isolates derived from OECM’1 cells were subjected to tryptic digestion, the peptides subjected to mass-spectroscopy and peptide families identified in an unbiased manner by comparison to a human peptide database using proprietary methods at Creative Proteomics, Inc. Data summarized in Figure 1D are in terms of arbitrary intensity units of respective peptide families. As a positive control, both duplicate samples revealed MxA/Mx1 peptides. Of the additional >200 peptide families in the dataset, unexpectedly, the BACH1 transcription factor family (comprising BACH1, BACH2, Nrf1, Nrf2, and Nrf3) yielded by far the strongest signal, followed by lesser and variable signals for other peptide families (Figure 1D).

### 3.2. Confirmation of Association of BACH1 and Nrf2 with GFP-MxA Condensates by Immunofluorescence Assays

**Figure 2:**
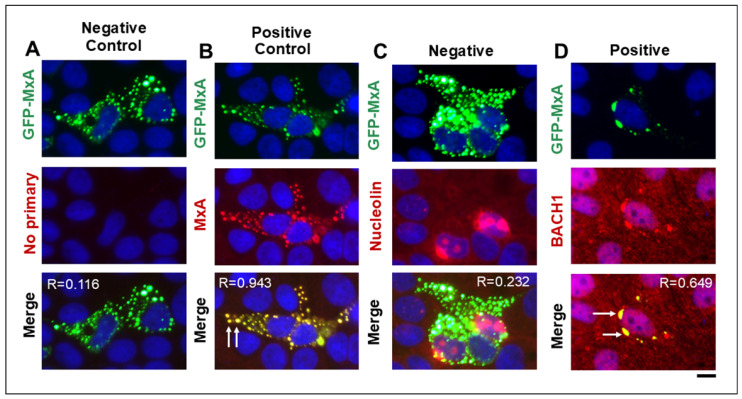
Immunofluorescence analyses for the association of some candidate proteins from Panel 1D with GFP-MxA condensates. OECM1 cultures expressing GFP-MxA were fixed two days after transfection and evaluated for the presence of nucleolin or BACH1 in the GFP-MxA condensates in immunofluorescence assays. Panels (**A**,**B**) represent negative and positive controls without primary pAb or with anti-MxA pAb. Panels (**C**,**D**) represent data for nucleolin and BACH1 respectively. White arrows point to GFP condensates containing MxA or BACH1 by immunofluorescence.

Immunofluorescence analyses of cells expressing GFP-MxA condensates were then carried out using respective antibodies to the top 6–8 candidate proteins (listed in Figure 1D) to help validate potential identifications [24,25,28,30]. Figure 2A illustrates a negative control without primary antibody, and Figure 2B (white arrows) illustrates a positive control verifying the presence of immunoreactive MxA (in red) in GFP-MxA condensates. Figure 2C shows a negative result for nucleolin, and Figure 2D (white arrows) shows a positive result for BACH1.

Figure 3A illustrates a negative control in which a rabbit pAb to BMPR1 (bone morphogenetic protein receptor 1) was used as an irrelevant antibody. Figure 3B (white arrows) reconfirms the detection of BACH1 in GFP-condensates. Since, from among the family members of these transcription factors enumerated in Figure 1D, Nrf2 is already known to be antiviral towards influenza A virus (IAV) as well as additional RNA viruses [37,38,40,41], we wondered whether Nrf2 might also colocalize with the GFP-MxA which is also antiviral towards IAV and several RNA and DNA viruses. The data in Figure 3C (white arrows) reveal that the Nrf2 transcription factor strongly colocalized with GFP-MxA condensates.

It is noteworthy that in Figure 2 and Figure 3, the same batch of secondary A594-tagged donkey anti-rabbit IgG was used in all the assays (in the negative controls, the positive MxA control, and in assays for the new candidate proteins). Similar immunoassays were negative for lamin, annexin A, nucleophosmin, plectin and histone species using respective pAb and mAb. Thus, this immunoassay-based survey confirmed the presence of immunoreactive MxA in GFP-MxA condensates (as positive control), and provided data to support the presence of BACH1 and Nrf2 immunoreactive peptides in such condensates (Figure 2 and Figure 3). Since BACH1 and Nrf2 transcription factors are well known for their repressor and activator functions respectively in regulating cellular responses to environmental and oxidative stresses, and in cancer progression and metastases, Refs. [31,32,33,34,35,36]), additional techniques were used to validate this association.

**Figure 4:**
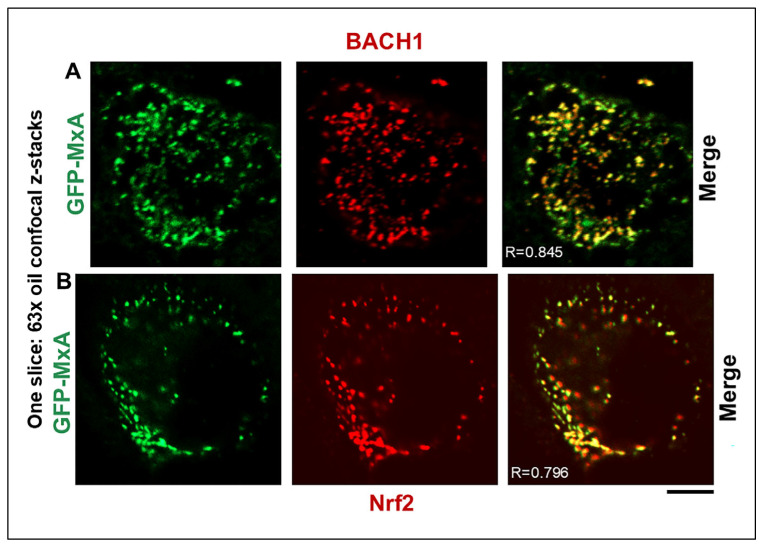
Confocal z-stack evaluation of the colocalization of BACH1 and Nrf2 with GFP-MxA condensates—evidence of heterogeneity. Panels (**A**,**B**), GFP-MxA condensates expressing OECM1 cells plated on cover-slip bottom plates were processed for detecting BACH1 or Nrf2 by immunofluorescence using respective rabbit pAbs. Imaging data were collected using a 63× oil objective; the figure illustrates one representative z-stack slice. Scale bar = 5 µm.

**Figure 5:**
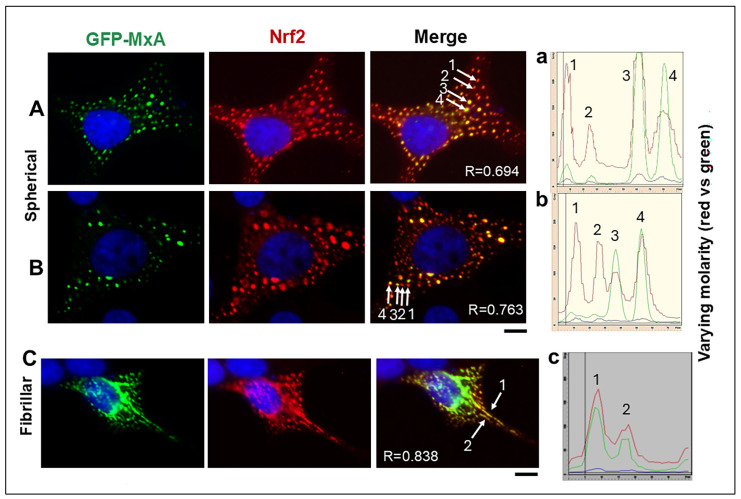
Heterogeneity of distribution of Nrf2 in GFP-MxA condensates. Wide-field immunoassay imaging of Nrf2 in OECM1 cells evidencing spheroidal or fibrillar phenotype of GFP-MxA condensates. Panels (**A**,**B**) show examples of cells with spheroidal GFP-MxA condensates revealing disparate amounts of Nrf2. The arrows in the “merge” panel highlight condensates (numbered 1 through 4) which were scanned linearly and their red and green pixel intensities represented in the insets (**a**,**b**) on the right. Panel (**C**), co-localization of Nrf2 with fibrillar GFP-MxA condensates; Condensates 1 and 2 scanned in inset (**c**) on the right.

Figure 4 illustrates one representative slice of a high-magnification z-stack collected using confocal microscopy, confirming the co-association of BACH1 and Nrf2 with GFP-MxA condensates. Curiously, while significant co-association between GFP-MxA and the respective BACH1 and Nrf2 antigenicity is clear (Pearson’s R = 0.845 and = 0.796 respectively), some variability in the relative overlap of red and green signals is clearly evident. This suggests that not all GFP-MxA condensates contain the same molar amounts of BACH1 or Nrf2. Distinctive variations in Nrf2 distribution were also evident using wide-field immunofluorescence microscopy (Figure 5(Aa,Bb)). The insets on the right highlight respective condensates 1 through 4 in Figure 5A,B, showing the differences between red and green pixels in each condensate. These data demonstrate critically that the distribution of BACH1 and Nrf2 is not always necessarily the same in all GFP-MxA condensates, even in the same cell; in contrast, in Figure 3B,C, it is evenly distributed. These data emphasize the biochemical heterogeneity of GFP-MxA condensates from cell to cell, and even among condensates in the same cell. Additionally, while Figure 5A,B focus on spheroidal GFP-MxA condensates, data in Figure 5C confirm the co-association of Nrf2 with fibrillar GFP-MxA.

### 3.3. Alternative Confirmation of the Association of GFP-BACH1 and GFP-Nrf2 with HA-MxA Condensates

**Figure 6:**
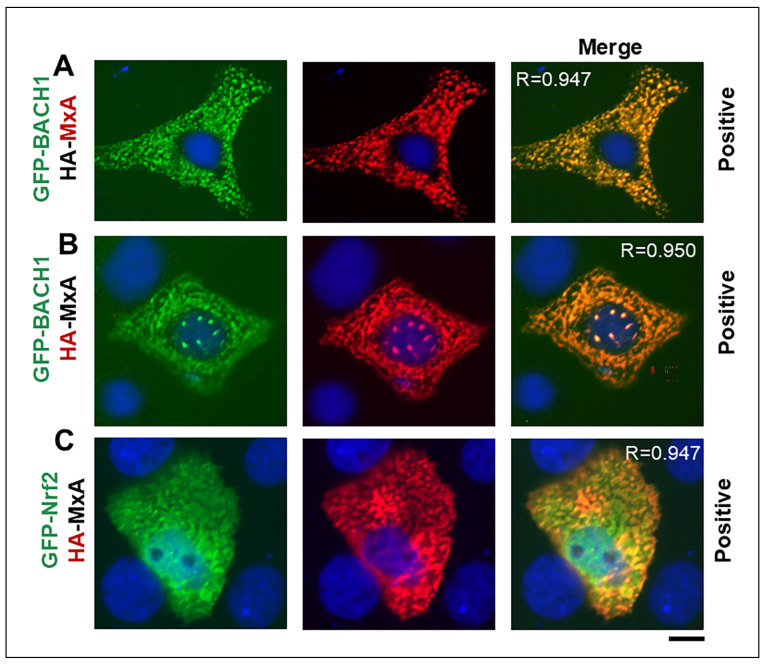
Alternative approach confirming the association of GFP-BACH1 and GFP-Nrf2 with HA-MxA. OECM1 cells were transiently transfected with pHA-MxA together with either pGFP-BACH1 or pGFP-Nrf2. Two days later, the cultures were fixed, and the transfected cells were imaged for respective GFP-tagged proteins (in green) and for HA-MxA using the anti-HA tag pAb (in red). (**A**,**B**) colocalization of HA-MxA with GFP-BACH1; (**C**), co-localization of HA-MxA with GFP-Nrf2.

The association of BACH1 and Nrf2 with MxA condensates was evaluated using an approach and reagents different from the immunoassays summarized in Section 3.2 above. OECM1 cells were transiently transfected with an expression vector for HA-MxA together with expression vectors for either GFP-BACH1 or GFP-Nrf2. Two days later, the cultures were fixed, and the transfected cells were imaged for respective GFP (in green) and for MxA using an anti-HA tag pAb. Thus, in these experiments, the imaging strategy and reagents are completely different from those in Figure 2, Figure 3, Figure 4 and Figure 5. Figure 6 summarizes representative data obtained using this alternative approach. Figure 6A,B confirm the colocalization of GFP-BACH1 with HA-MxA condensates, while Figure 6C confirms the colocalization of GFP-Nrf2 with HA-MxA condensates.

### 3.4. Biochemical Interaction Studies Using Western Blots

**Figure 7:**
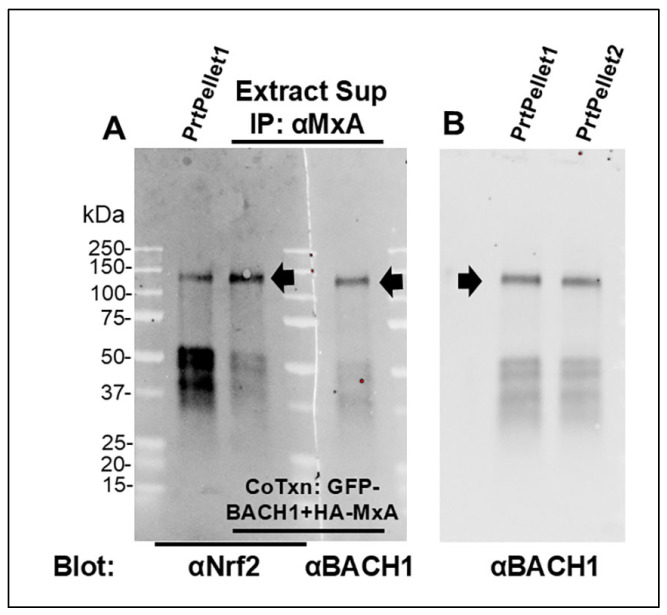
Western blot detection of full-length Nrf2 and BACH1 proteins (black arrowheads) in Protein A-Dynabead-immunoisolates using anti-MxA pAb after SDS-PAGE (4–15% gradient gels). In Panels (**A**,**B**), PrtPellet 1 and 2 represent aliquots of Dynabead-based isolates of extracts of OECM1 cells prepared as in Figure 1D (OECM1 cells were transfected with pGFP-MxA and treated with IFN-λ1 for 2 days, followed by prefixation, sonication, clearance of debris and immunoisolation of supernatant using anti-MxA pAb). In Panel (**A**), the two central lanes (black arrowheads) show anti-MxA pAb immunoisolation from extracts prepared from OECM1 cells co-transfected with GFP-BACH1 and HA-MxA vectors for 2 days (as in Figure 6A). The blots were probed using the respective anti-Nrf2 or anti-BACH1 pAbs as indicated. Note that, as previously known [45,46,47], the apparent mobility of Nrf2 and BACH1 in SDS-PAGE is slower (>100 kDa) than the size predicted from the amino acid sequence (approx. 60–65 kDa).

Western blot assays were carried out to verify whether the Protein A Dynabead-immunoisolated protein pellets used in the analyses in Figure 1D did indeed contain full-length endogenous BACH1 and full-length endogenous Nrf2 proteins. Thus, respective aliquots of the protein pellets from GFP-MxA expressing cells isolated using anti-MxA pAb (PrtPellet1 and PrtPellet2), similar to those used to derive the data in Figure 1D, were probed for Nrf2 (Figure 7A, left-most lane) or for BACH1 (Figure 7B, both lanes) by Western blotting. The data in Figure 7A,B confirm the presence of full-length Nrf2 and BACH1 proteins in these MxA-condensate isolates, validating the discovery of corresponding peptides in such isolates as in Figure 1D.

The strong colocalizations observed between HA-MxA on the one hand and either GFP-BACH1 or GFP-Nrf2 on the other hand in the experiments summarized in Figure 6 (note that Pearson’s R were approximately 0.95 in all “merge” panels), suggested that this co-transfection approach (preparing extracts from cells transiently co-transfected with vectors of respective full-length tagged constructs) might be useful for protein interaction studies. Figure 7A (right two lanes, arrowheads) shows evidence that anti-MxA pAb cross-isolated Nrf2 as well as BACH1 from a prefixed extract of GFP-BACH1- and HA-MxA-cotransfected cells. Note that the middle lane in Figure 7A shows that endogenous Nrf2 was indeed pulled out using anti-MxA pAb (as was BACH1 also), indicating that MxA, Nrf2 and BACH1 were present in the same complex. These data consolidate the evidence derived from the immunofluorescence assays shown in Figure 2, Figure 3, Figure 4, Figure 5 and Figure 6.

**Figure 8:**
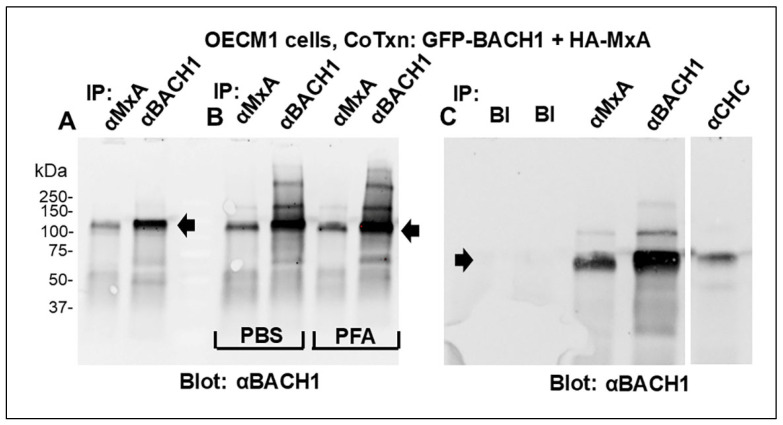
HA-MxA interacts with a subset of cellular BACH1 even without PFA prefixation. Extracts prepared from OECM1 cells transiently expressing GFP-BACH1 and HA-MxA for 2 days were prepared with PFA prefixation [Panels (**A**,**B**) (PFA lanes) and (**C**)] or without fixation (Panel (**B**), “PBS” lanes). Matching aliquots (within Panels (**A**), (**B**) and (**C**) respectively) were subjected to Protein A-Dynabead pullout using the indicated pAbs or without pAb (Bl) or an irrelevant pAb (to clathrin heavy chain, CHC). The respective pullout isolates were subjected to SDA-PAGE (4–15%) and Western blotting as indicated. Arrowheads indicate full-length BACH1 protein.

Side-by-side comparisons of immunopullout assays and Western blots using extracts of co-transfected OECM1 cells suggest that MxA interacts with a subset of cellular BACH1. In Figure 8A,C, the amounts of BACH1 in the respective anti-MxA pullout lanes are lower than in the anti-BACH1 lanes. Curiously, this interaction between MxA and BACH1 did not require the prefixation of cells with PFA. In Figure 8B, the extent of BACH1 pullout using anti-MxA pAb was comparable in cells prefixed with PFA or kept in PBS alone. Figure 8C, compares the anti-MxA and anti-BACH1 pullout to controls without added primary pAb or with the irrelevant anti-CHC pAb. Taken together, the data in Figure 7 and Figure 8 validate the presence of full-length BACH1 and Nrf2 proteins in association with MxA.

**Figure 9:**
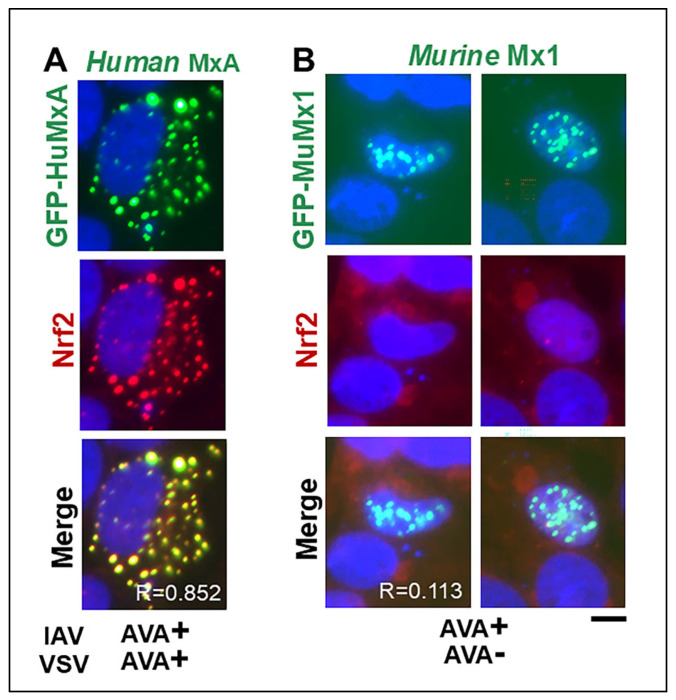
Selective association of Nrf2 with human GFP-MxA cytoplasmic condensates but not with murine GFP-Mx1 nuclear condensates. Human OECM1 cells were transiently transfected with vectors for human GFP-MxA or murine GFP-Mx1 to generate the respective cytoplasmic or nuclear condensates in 2 days [27]. The cultures were fixed and immunoassayed for Nrf2 using the same primary and secondary antibodies. Panel (**A**) shows accumulation and colocalization of endogenous Nrf2 with cytoplasmic condensates of human GFP-MxA. Panel (**B**) shows an absence of Nrf2 in nuclear condensates of murine GFP-Mx1. Divergent antiviral activity (AVA) +ve or −ve against IAV (influenza A virus) or VSV (vesicular stomatitis virus) when assayed in A549 cells [27].

Human MxA is a cytoplasmic protein that generates a cytoplasmic GFP-MxA condensate [48,49,50,51,52,53,54,55]. In contrast, its murine ortholog Mx1 is a nuclear protein and we have shown previously that murine GFP-Mx1 generates nuclear structures with properties of condensates [27]. Since endogenous Nrf2 provided strong data showing immunofluorescence colocalization with human GFP-MxA condensates in the cytoplasm (Figure 3C and Figure 9A), we investigated whether transcription factor Nrf2 might also associate with murine Mx1 nuclear condensates. For this evaluation, a vector for murine GFP-Mx1 was transfected into human OECM1 cells, and the cells were allowed to form nuclear GFP-Mx1 condensates over 2 days. The presence of Nrf2 in such condensates was then evaluated in immunoassays. The data in Figure 9B show the absence of colocalization between murine GFP-Mx1 nuclear condensates and endogenous Nrf2. Thus, the association of Nrf2 with Mx family members is selective for the human GFP-MxA condensate.

### 3.5. Association of the Redox-Protective Enzyme Heme Oxygenase 1 (HO1) but Not p62/Sequestosome 1 with GFP-MxA Condensates

**Figure 10:**
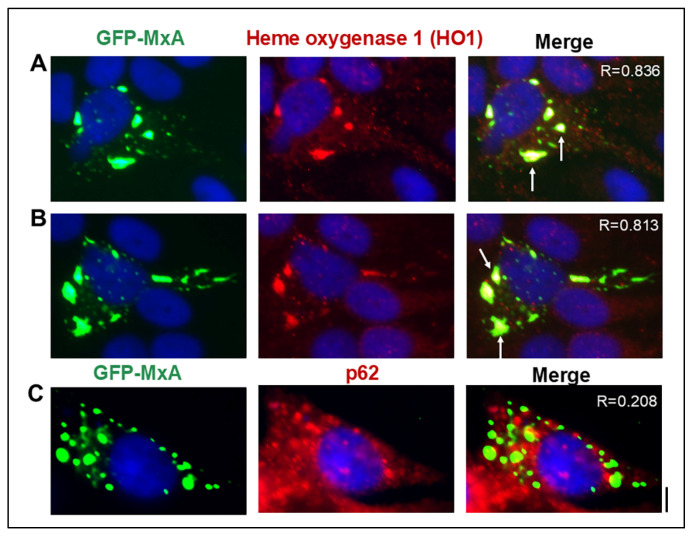
Association of heme oxygenase 1 (HO1) with cytoplasmic GFP-MxA condensates (white arrows) (Panels (**A**,**B**)), but not p62 (Panels (**C**)). OECM1 cells evidencing cytoplasmic condensates of GFP-MxA, were immunoassayed for HO1 or p62 using respective pAbs.

Both BACH1 and Nrf2 regulate the expression of genes involved in the oxidative stress response—BACH1 is typically a transcriptional repressor while Nrf2 is a transcriptional activator [31,32,33,34]. Heme Oxygenase 1 (HO1), an oxidative-stress protective protein with antiviral activity [2,34,39], is the downstream product of a target gene regulated up (by Nrf2) or down (by BACH1) through a balance between these two transcription factors. Moreover, HO1 itself displays antiviral activity towards RNA viruses and mediates functions of innate immunity [34,38,39]. Thus, we investigated whether HO1 might be associated with GFP-MxA condensates in cells that also display strong Nrf2 (Figure 3C and Figure 9A). The immunoassay data in Figure 10A,B revealed the association of the HO1 with GFP-MxA cytoplasmic condensates with R > 0.8.

Nrf2 is known to be sequestered in the cytoplasm in condensates of p62/sequestosome-1 referred to as “p62 bodies” [38,45,46,47]. The protein p62 serves as a selective autophagy receptor that binds ubiquitinated proteins for subsequent degradation, including as part of the antioxidant stress response. Thus, we investigated whether GFP-MxA condensates might represent such p62 bodies. The data in Figure 10C show that GFP-MxA condensates (which we show above in Figure 2, Figure 3, Figure 4, Figure 5, Figure 6, Figure 7, Figure 8, Figure 9 and Figure 10 to include Nrf2, BACH1 and XO1) did not include p62 (R only 0.2). Thus, MxA condensates do not represent p62 bodies. Additionally, GFP-MxA condensates were negative for KEAP1 and Cul3.

### 3.6. Dynamic Tonicity-Driven Regulation of Nrf2 Transcription Factor Condensates in Oral Cancer Cells

**Figure 11:**
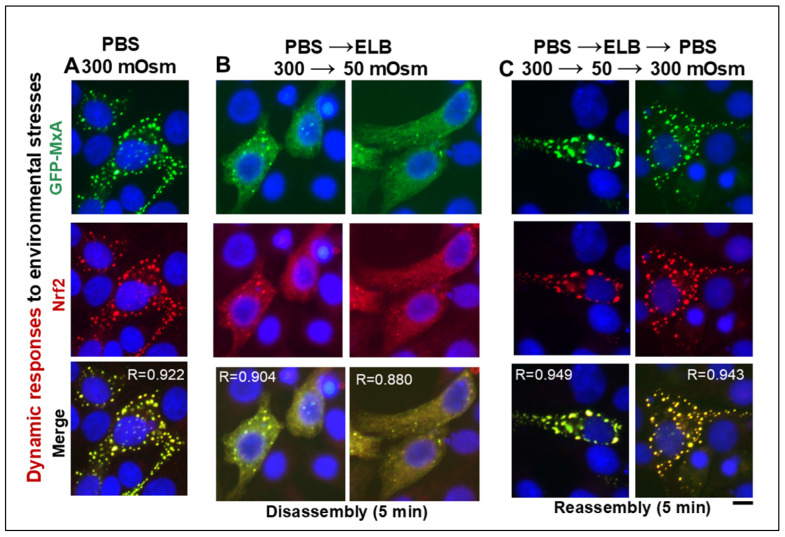
Tonicity-driven rapid disassembly and reassembly of endogenous Nrf2 together with GFP-MxA condensates in oral cancer cells. Nrf2 structures (imaged in red) in OECM1 cells expressing GFP-MxA (imaged in green) following exposure of cultures to buffers of different tonicity (at 37 °C) for the indicated times were evaluated following fixation and immunofluorescence imaging. Panel (**A**), culture in PBS (phosphate-buffered saline, 300 mOsm) for 5 min, then fixation in 4% PFA in PBS. Panel (**B**), culture in PBS for 5 min, shift to ELB (erythrocyte lysis buffer, 50 mOsm) for 5 min and fixation in 4% PFA in ELB. Panel (**C**), culture initially in PBS, then in ELB for 5 min, followed by a shift to PBS for 5 min and then fixation in 4% PFA in PBS.

In the “exposome” hypothesis [3,4,25], we posit that in the absence of overt causes of oral cancer (such as tobacco, betel-nut chewing or alcohol consumption), the exposure of the floor of the mouth (the “high-risk region”) to environmental stresses of tonicity, temperature and pH (for example hypotonicity due to saliva and to beverages such as water, tea or coffee) sets into motion repeated cycles of condensate disassembly and reassembly. We envision that pro-carcinogenic transcription factors in cells subjected to such cycling over long periods of time (months or years) might, on occasion, lead to aberrant signaling, triggering a cancer initiation event. With the present discovery of the association of the positive transcription factor Nrf2 with cytoplasmic condensates in oral cells, we investigated whether Nrf2-positive structures in such cells might display dynamic disassembly and reassembly. In these experiments (as in Figure 11), disassembly and then reassembly of GFP-MxA into new condensates helped track the process. The data in Figure 11B show the rapid cytoplasmic dispersal of both GFP-MxA and transcription factor Nrf2 in cells exposed to beverage-like hypotonicity (ELB at 50 mOsm) for 5 min. Remarkably, both Nrf2 and GFP-MxA reassembled together into new condensates when cells were subsequently exposed to isotonic medium. Taken together, the data in Figure 11 provide a foundation for the possibility that assorted pro-carcinogenic transcription factors can indeed cycle in and out of cellular condensates in oral cells subjected to environmental stresses. The question of whether repeated recycling of pro-carcinogenic transcription factors out of and then back into condensates in this manner might lead to aberrant signaling and trigger carcinogenesis is intriguing.

### 3.7. Sulforaphane (SFN) Disperses GFP-MxA and Nrf2 into the Cytosol

**Figure 12:**
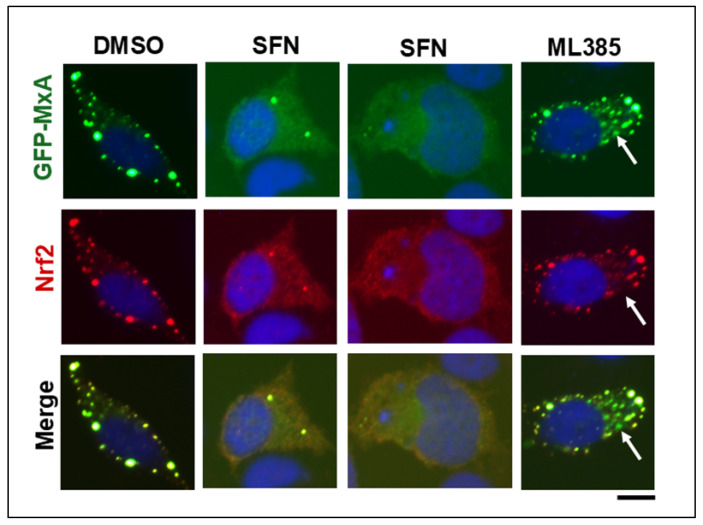
Sulforaphane (SFN) disassembles GFP-MxA- and Nrf2-containing condensates. OECM1 cultures transiently expressing GFP-MxA for 2 days were exposed to DMSO, SFN (175 µM) or ML385 (50 µM) for 4 h and fixed using 4% paraformaldehyde. GFP-MxA (in green) and Nrf2 (in red) were imaged as indicated in the legend in Figure 11 (n = 10–20 per variable). The figure illustrates representative images. White arrows indicate GFP-MxA condensates with weak Nrf2 staining.

The small molecules sulforaphane (SFN) and ML385 have often been used to enhance or inhibit the activity of Nrf2 respectively [34,35,36,37,38,40,41]. The data in Figure 12 show that SFN caused the disassembly of GFP-MxA condensates with the release of both GFP-MxA and Nrf2 into the cytoplasm. The lack of KEAP1 in GFP-MxA condensates (see above) suggests that the mechanism underlying this dispersal is KEAP1-independent. GFP-MxA condensates were preserved in ML385-treated cells but with an increase in heterogeneity of Nrf2 among the condensates (white arrows in Figure 12 highlight GFP-MxA condensates with little Nrf2).

## 4. Discussion

An unbiased proteomics-based evaluation of peptides associated with cytoplasmic GFP MxA condensates isolated from human oral cancer cells revealed the unexpected presence of peptides of the BTB and b-Zip transcription factor family, with the identification of the BACH1 protein family in the condensate immunoisolates. Two members of this protein family—the transcriptional repressor BACH1 and the transcriptional activator Nrf2—have been investigated extensively in the past two to three decades for their roles in cellular responses to oxidative and environmental stresses, antiviral defense and innate immunity, and cancer progression and metastasis [31,32,33,34,35,36]. Thus, with this initial peptide-family identification, we focused on validating the possible association of full-length BACH1 and Nrf2 proteins with GFP-MxA condensates. Respective immunofluorescence assays of oral cancer cells expressing GFP-MxA condensates, Western blots including cross-immunopullout experiments, and studies with full-length GFP-BACH1 and GFP-Nrf2 coexpressed in cells together with HA-MxA validated the co-association of these proteins with the same condensates. Moreover, unexpectedly, HO1, the downstream product of the activation of Nrf2, was also included in GFP-MxA condensates. Thus, the inclusion of human MxA, BACH1, Nrf2 and HO1 defines a novel class of “antioxidant” cytoplasmic condensates. These structures were distinct from “p62 bodies,” which comprise the p62/sequestome-1 protein that binds Ub-Nrf2, targeting Nrf2 towards autophagic degradation [38,45,46,47] and were also negative for KEAP1 and Cul3. Moreover, in a further distinction, murine Mx1, which forms nuclear condensates, did not associate with Nrf2.

Nevertheless, the foundational antiviral activity that led to the recognition starting in 1963 of nuclear murine Mx1 and in the 1970s of its ortholog cytoplasmic human MxA was the inhibition of influenza A virus replication (IAV) [48,49,50,51,52,53,54]. This antiviral repertoire of Mx family members has since been expanded to include an extensive collection of RNA- and DNA-containing viruses with some distinctions (for example, the cytoplasmic MxA shows antiviral activity against the rhabdovirus VSV, but the nuclear Mx1 does not [27,48,49,50,51,52,53,54]. In cell-free assays, MxA inhibits early RNA viral transcription through a mechanism not yet completely elucidated [52,53,54]. Amazingly, Nrf2 also inhibits IAV replication at an early stage of infection [37,38,40,41]. Moreover, Nrf2 also displays antiviral activity towards additional RNA and DNA viruses such as respiratory syncytial virus (RSV), SARS-CoV-2, herpes simplex 1 and vaccinia virus [37,38,40,41]. Even HO1 displays antiviral activity towards RNA viruses [38,39]. Thus, taken together, the literature points to the possibility of multiple antiviral mechanisms mediated jointly by the components of the MxA, Nrf2, and HO1-containing “antioxidant condensate”. In the case of MxA, it is the dispersed phase that appears to possess antiviral activity, with the condensate representing a storage depot [25,55].

The balance between the cytoplasmic and nuclear pools of Nrf2 and BACH1 regulates their respective transcriptional activities [31,32,33,34,35,36,56]. Increased cellular BACH1 is associated with increased metastasis of triple-negative breast cancer, and increased Nrf2 with hepatocellular carcinoma [57,58]. p62 condensates incorporate Ub-Nrf2 and target it towards degradation—mutations in p62 and associated proteins represent gain-of-function changes which enhance Nrf2 function in carcinogenesis [45,46,47].

While human MxA is known to be expressed in healthy gingival/buccal epithelium, likely through induction by IFN-λ species or α-defensins [29], in the present study, we used GFP-MxA as a reporter for studies of condensate dynamics in oral cancer cells. In previous experiments, we had observed that cytoplasmic GFP-MxA condensates in oral epithelial cells were rapidly (within 1–3 min) dispersed by saliva- and beverage-like hypotonicity (range 30–100 mOsm). This then triggered an active cellular process which mediated a “spontaneous” reassembly of MxA into a new set of condensates in the next 5–10 min [25]. This process reflected water influx and “uncrowding” of the cytoplasm in the disassembly phase, and water efflux and “recrowding” of the cytoplasm in the reassembly process [25,28,30]. This process was temperature-sensitive (faster at 50 °C and slower at 5 °C; Ref. [25]) and involved the WNK-SPAK/OSR1 kinase pathway, which regulates chloride and water influx and efflux [25]. The discovery that the transcription factors BACH1 and Nrf2 associate with MxA condensates suggested that these transcription factors might also be regulated by water and chloride influx and efflux in oral epithelial cells. Indeed, the present data (Figure 11) show that Nrf2 disassembles in oral cancer cells in response to hypotonicity and reassembles into new condensates when cells are exposed to isotonic conditions. These data provide a basis for suggesting that hypotonicity, temperature and environmental stresses preferentially inflicted on cells in the floor of the mouth along the path of liquid transit might particularly disrupt the normal function of Nrf2, BACH1 and HO1 in this location in the mouth. We posit this mechanism as a possible basis for this region in the mouth for representing a “high-risk zone” for cancer development. Remarkably, very recently, Cook et al. [59] reported that even mild hypotonicity (half-strength plasma corresponding to 170 mOsm) drives global transcriptomic reprogramming in murine macrophages mimicking the response to Type I interferon signaling.

## 5. Conclusions

All of us repeatedly challenge our oral mucosa with the stresses of tonicity and temperature every single day. We posit that these stresses trigger repetitive cycles of disassembly/reassembly of biomolecular condensates in oral epithelial cells in the U-shaped high-cancer-risk region in the mouth along the path of liquid transit (the “exposome”) [3,4]. The association of transcription factors BACH1, Nrf2 and the protective protein HO1 with MxA condensates in such cells defines a novel “antioxidant condensate” in such cells. The data suggest a novel subcellular mechanism for the initiation of oral cancer in the “high-risk” region—the repetitive condensate disassembly and reassembly cycles of these prooncogenic transcription factors resulting in aberrant episodic cancer initiation events.

## Data Availability

The original contributions presented in this study are included in the article. Further inquiries can be directed to the corresponding author.
